# Compressed sensing-based image reconstruction for discrete tomography with sparse view and limited angle geometries

**DOI:** 10.1371/journal.pone.0327666

**Published:** 2025-07-11

**Authors:** Haytham A. Ali, Essam A. Rashed, Hiroyuki Kudo

**Affiliations:** 1 Institute of Systems and Information Engineering, University of Tsukuba, Tsukuba, Japan; 2 Department of Mathematics, Faculty of Science, Sohag University, Sohag, Egypt; 3 Graduate School of Information Science, University of Hyogo, Kobe, Japan; Islamia University of Bahawalpur: The Islamia University of Bahawalpur Pakistan, PAKISTAN

## Abstract

This paper addresses the image reconstruction problem in discrete tomography, particularly under challenging imaging conditions such as sparse-view and limited-angle geometries commonly encountered in computed tomography (CT). These conditions often result in low-quality reconstructions due to insufficient projection data and incomplete angular coverage. To overcome these limitations, we propose a novel reconstruction framework that integrates compressed sensing (CS) with a parametric level set (PLS) method tailored for discrete images. The proposed approach leverages prior knowledge of discrete gray-level values and employs a parametric level set function to represent boundaries in both binary and multi-gray-level images. Unlike previous methods, our PLS is constructed using a dictionary of basis functions composed of single-scale or multiscale Gaussian functions. Reconstruction is formulated as 𝚤_1_-norm minimization of Gaussian coefficients, promoting sparsity. We assess the method’s robustness by introducing varying levels of Gaussian noise into the projection data under both sparse-view and limited-angle conditions. Quantitative evaluations using PSNR, SSIM, and Dice coefficients demonstrate that the proposed method preserves boundary sharpness and accurately reconstructs discrete intensity levels, even in highly undersampled and noisy scenarios. Simulations and experiments on both synthetic and real CT data confirm that the proposed approach consistently outperforms conventional methods in terms of reconstruction quality, boundary accuracy, and noise robustness.

## Introduction

Tomography is a widely used imaging technique in fields such as medical diagnostics, industrial inspection, astrophysics, archaeology, and materials science [1–3]. It relies on collecting projection data of an object from multiple angles to reconstruct its internal structure. However, in many practical scenarios, obtaining complete projection data is infeasible due to time, cost, or physical constraints. This leads to reconstruction problems involving sparse-view or limited-angle geometries, which are particularly ill-posed and prone to artifacts.

Discrete tomography, a subset of tomography, focuses on reconstructing images composed of a small number of known gray levels [4–6]. This setting arises frequently in applications such as industrial CT of manufactured parts, micro-CT of bone, or electron tomography of nanoparticles [7–11]. Although incorporating prior knowledge of discrete intensity levels can significantly enhance reconstruction quality, most conventional algorithms—such as filtered backprojection (FBP) [12–15] and standard algebraic reconstruction techniques (ART) [16–19]—do not effectively exploit this information, particularly under limited or noisy data conditions.

Compressed sensing (CS) theory [20–22] offers a powerful framework for recovering sparse signals from undersampled measurements. Several studies have applied CS to CT reconstruction [23–25], but its success heavily depends on the assumed sparsity prior and the chosen regularization strategy. In discrete tomography, where the target images are piecewise constant with well-defined boundaries and a limited set of intensity levels, standard CS formulations often struggle to preserve edge sharpness and enforce discrete values, particularly in the presence of noise.

Parametric level set (PLS) methods have been introduced to represent object boundaries using compact basis functions [26, 27]. These methods are effective for shape-based inverse problems and have been extended to tomography [28, 29]. However, existing PLS approaches face challenges when modeling multi-region or multi-level images, especially under data-limited scenarios [26, 30, 31]. Furthermore, prior PLS methods typically rely on predefined shape dictionaries or simple basis functions that lack flexibility in capturing fine boundary variations.

**Fig 1:**
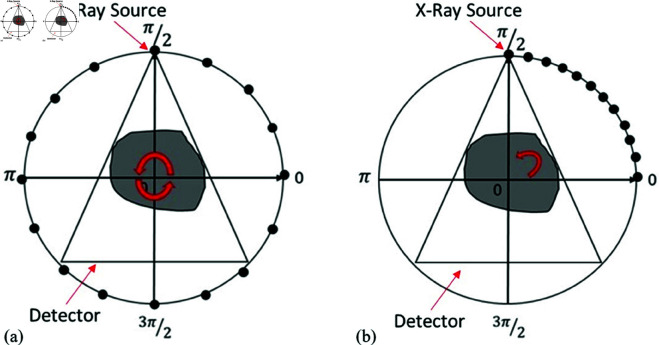
Diagram of limited-data tomography collected through (a) sparse-view CT scan [0,2π] and (b) limited-angle CT scan [0,π/2].

To address these limitations, we propose a novel reconstruction method tailored to discrete tomography under sparse-view and limited-angle conditions, as shown in Fig 1. We introduce a model-based method that integrates compressed sensing (CS) theory with a parametric level set (PLS) formulation. The key idea is to use a dictionary of Gaussian basis functions-either single-scale or multiscale-distributed over the image domain to represent the object’s shape and boundaries. By enforcing sparsity through 𝚤_1_-norm regularization of the basis coefficients and leveraging prior knowledge of discrete intensity levels, the method enhances reconstruction quality in sparse-view and limited-angle geometries. Unlike conventional methods, our technique is designed specifically for discrete tomography and excels at preserving boundary accuracy and robustness to noise. Practical experiments, including PSNR, SSIM , and Dice coefficients trends under varying noise levels and projection setups, demonstrate that our method achieves higher reconstruction quality, with improved preservation of fine details, even under sparse and noisy data conditions. These results confirm that the proposed approach outperforms previous techniques and other reconstruction methods.

The remainder of this paper is organized as follows: the Background section reviews the fundamentals of compressed sensing and the OWL-QN optimization method. The Parametric Level Set Method section introduces the proposed reconstruction model based on parametric level sets. The Basis Function section discusses implementation aspects, including basis function design. The Experimental Studies section presents simulation and experimental results. Finally, the Conclusion section summarizes our contributions and findings.

## Background

To make this paper self-contained, this section offers a brief overview of compressed sensing theory and the orthant-wise limited-memory quasi-Newton (OWL-QN) algorithm, which is employed to address optimization problems involving 𝚤_1_-norm regularization.

### Compressed sensing theory

The concept of compressed sensing (CS), introduced by Donoho and Candès [20, 21], posits that it is possible to recover the original signal from a sparse set of data samples. This has significant implications for computed tomography (CT), where projection data may be incomplete due to physical or practical limitations [20]. CS offers a substantial advantage over traditional Nyquist sampling methods by providing a lower bound for sampling requirements. In CT applications, the projection process can be mathematically described as a linear system:

y=Ax (1)

where $y\in {\mathbb{R}}^{M}$ represents the observed data, $A\in {\mathbb{R}}^{M\times N}$ is the measurement sampling matrix, and $x\in {\mathbb{R}}^{N}$ is a sparse vector containing the pixel values of the reconstructed image, with M≪N.

The objective is to recover the original signal *x* from the observed data by solving the following 𝚤_0_-norm optimization problem:

$$\min\|x{\|}_{0}\;\text{subject to}\;y=Ax$$ (2)

However, solving the 𝚤_0_-norm optimization problem is known to be NP-hard. Consequently, Eq 2 is often approximated by converting it into an 𝚤_1_-regularized optimization problem:

$$\min\|x{\|}_{1}\;\text{subject to}\;y=Ax$$ (3)

This can be further reformulated as:

$$\min\frac{1}{2}\|y-Ax{\|}_{2}^{2}+\lambda \|x{\|}_{1}$$ (4)

where λ>0 is the regularization parameter. The choice of λ is critical to the reconstruction process. In this study, we address this by solving Eq 4 for several small values of λ and evaluating the quality of the resulting reconstructions. By comparing these results, we identify the value of λ that yields the most optimal reconstruction.

In the context of CT image reconstruction, sparsity is not directly present in the image domain but can be effectively induced in the parametric level set framework, where image boundaries are encoded using a sparse set of basis functions. By modeling the level set function as a linear combination of Gaussian basis functions and enforcing sparsity on their coefficients through 𝚤_1_-norm minimization, we cast the reconstruction task as a compressed sensing problem tailored to discrete tomography.

However, classical CS-based reconstruction methods are known to face challenges such as sensitivity to noise, loss of fine structural detail, and instability under high undersampling conditions [22–24, 32]. To mitigate these issues, our approach incorporates prior knowledge of discrete gray levels and employs a structured dictionary of basis functions. This design promotes stability, reduces artifacts, and improves boundary preservation under both sparse-view and limited-angle acquisition scenarios.

To solve the 𝚤_1_-regularized problem efficiently, we employ the Orthant-Wise Limited-memory Quasi-Newton (OWL-QN) algorithm, which is described in the following section [33].

### OWL-QN method

A key challenge in solving Eq 4 is that the 𝚤_1_-norm is not differentiable at zero. To address this, Andrew and Gao [33] proposed the OWL-QN (Orthant-Wise Limited-memory Quasi-Newton) algorithm, specifically designed to solve the 𝚤_1_-regularized optimization problem of the form:

$$\min_{x\in {\mathbb{R}}^{n}}\;f(x)=l(x)+C\|x{\|}_{1}$$ (5)

In this formulation, $l:{\mathbb{R}}^{n}\to \mathbb{R}$ represents the smooth data fidelity term, which is continuously differentiable and bounded below, and *C*>0 is a regularization parameter that controls the strength of the sparsity penalty.

The OWL-QN algorithm leverages the insight that the 𝚤_1_-norm is differentiable within any given orthant—an orthant is a set of points in which each coordinate never changes sign, making the 𝚤_1_-norm a linear function of its argument. Consequently, the second-order behavior of the objective function *f* in a particular orthant is predominantly determined by the loss component l(x).

Geometrically, OWL-QN addresses the non-differentiability of the 𝚤_1_-norm by restricting the search direction to remain within a single orthant of the parameter space—that is, a region where the sign of each component of the vector remains fixed. Within each orthant, the 𝚤_1_-norm behaves as a linear function, avoiding the non-differentiable kink at zero. By projecting updates back into the same orthant, the algorithm avoids non-differentiable points in the optimization landscape and ensures smooth progress guided by the differentiable loss function. This orthant-wise strategy allows OWL-QN to maintain both numerical stability and sparsity in the solution. To take advantage of this, the algorithm constructs a quadratic approximation of the function *l*(*x*) within the orthant containing the current point. The search for the optimal solution is then confined to this orthant, along the direction of the minimum of the approximation.

To implement this, the algorithm uses a projection operator π(x;y), which constrains *x* to remain in the same orthant as *y*, defined component-wise as:

$$\pi_{i}(x_{i};y_{i})=\left\{ \begin{array}{cc} x_{i}, & \text{if}\;\text{sign}(x_{i})=\text{sign}(y_{i})\\ 0, & \text{otherwise} \end{array}\right.$$ (6)

where sign(·) is the sign function, with sign(0)=0.

Additionally, selecting which orthant to explore involves evaluating the pseudo-gradient ∇f(x) of the objective function:

$$(\nabla f{)}_{i}(x)=\left\{ \begin{array}{cc} \frac{\partial l(x)}{\partial x_{i}}+C\cdot \text{sign}(x_{i}), & \text{if}\;x_{i}\neq 0\\ \frac{\partial l(x)}{\partial x_{i}}+C, & \text{if}\;x_{i}=0\text{ and}\;\frac{\partial l(x)}{\partial x_{i}}<-C\\ \frac{\partial l(x)}{\partial x_{i}}-C, & \text{if}\;x_{i}=0\text{ and}\;\frac{\partial l(x)}{\partial x_{i}}>C\\ 0, & \text{otherwise} \end{array}\right.$$ (7)

The update formula for the OWL-QN algorithm is given by:

$$x^{\left(k+1\right)}=\pi_{\xi^{k}}\left(x^{k}+\alpha^{k}p^{k}\right)$$ (8)

where $\alpha^{k}$ is the step size, and $\xi_{i}^{k}\in {\mathbb{R}}^{n}$ indicates the reference orthant direction:

$$\xi_{i}^{k}=\left\{ \begin{array}{cc} \text{sign}(x_{i}^{k}), & \text{if}\;x_{i}^{k}\neq 0\\ \text{sign}(v_{i}^{k}), & \text{if}\;x_{i}^{k}=0 \end{array}\right.$$ (9)

Here, the vector $v^{k}=-\nabla f(x^{k})$ is the negative pseudo-gradient. The search direction ${\text{p}}^{\text{k}}$ is obtained via:

$$p^{k}=\pi_{v^{k}}\left({ℋ}^{k}v^{k}\right)$$ (10)

where ${ℋ}^{k}$ is the limited-memory BFGS (L-BFGS) approximation of the inverse Hessian of *l*(*x*) [34].

## Parametric level set method

**Fig 2:**
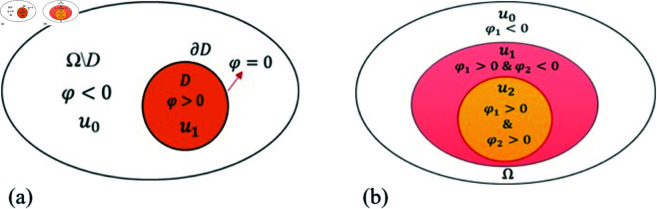
Illustration of zero-level set boundaries and multi-region level set modeling. (a) The zero-level set curve defines the boundary between the object and the region of interest. (b) Two level sets are used to model three regions, each indicated by a unique sign combination of $\varphi_{1}$ and $\varphi_{2}$.

In this paper, we employ the Parametric Level Set (PLS) method to tackle reconstruction problems of interest. In shape-based inverse problems, any domain Ω can be partitioned into two regions: the foreground (object) *D* and the background Ω ⧵ *D*, as illustrated in Fig 2a. Consequently, a model with two regions can be represented as follows:

$$u(x)=\left\{ \begin{array}{cc} u_{1}, & \text{if}\;x\in D\\ u_{0}, & \text{if}\;x\in \Omega ⧵D \end{array}\right.$$ (11)

Using the level set function φ(x), the model *u*(*x*) can be expressed as:

$$u(x)=u_{0}\left(1-H(\varphi (x))\right)+u_{1}H(\varphi (x)),$$ (12)

where H(·) denotes the Heaviside function, approximated smoothly as suggested in [27]:

$$H(x)=\left\{ \begin{array}{cc} 1, & \text{if}\;x>\epsilon \\ 0, & \text{if}\;x<-\epsilon \\ \frac{1}{2}+\frac{x}{2\epsilon }+\frac{1}{2\pi }\sin\left(\frac{\pi x}{\epsilon }\right), & \text{if}\;|x|\leq \epsilon \end{array}\right.$$ (13)

where [−ϵ,ϵ] is a small interval over which the function is smoothed [27].

Instead of using the signed distance function in the traditional level set method, we used the parametric form of the level set function, known as the parametric level-set (PLS), which can be expressed as:

$$\varphi (x,\alpha )=\left\{ \begin{array}{cc} \varphi (x,\alpha )>c, & \text{if}\;x\in D\\ \varphi (x,\alpha )=c, & \text{if}\;x\in \partial D\\ \varphi (x,\alpha )<c, & \text{if}\;x\in \Omega ⧵D \end{array}\right.$$ (14)

where *c* represents a small positive value. Using PLS, we can rewrite Eq 12 as:

$$u(x,\alpha )=u_{0}\left(1-H(\varphi (x,\alpha )-c)\right)+u_{1}H(\varphi (x,\alpha )-c).$$ (15)

Based on prior research by Aghasi *et al*. [27] and our own studies [35, 36], the parametric level set (PLS) function can be represented as a weighted sum of basis functions:

$$\varphi (x,\alpha )=\sum_{i=1}^{N}\alpha_{i}M_{i}(x)$$ (16)

where *N* denotes the number of basis functions, α is the PLS parameter vector, and *M*_*i*_(*x*) are the basis functions in the set $M=\{m_{1},m_{2},\ldots ,m_{N}\}$.

For scenarios involving more than two regions, as shown in Fig 2b with three regions, we need to employ two level set functions. The model *u*(*x*) can then be expressed as:

$$u(x,\alpha )=u_{0}\left(1-H(\varphi_{1}-c)\right)+u_{1}H(\varphi_{1}-c)\left(1-H(\varphi_{2}-c)\right)+u_{2}H(\varphi_{1}-c)H(\varphi_{2}-c).$$ (17)

Or more concisely:

$$u(x,\alpha )=u_{0}+(u_{1}-u_{0})H(\varphi_{1}-c)+(u_{2}-u_{1})H(\varphi_{1}-c)H(\varphi_{2}-c).$$ (18)

The *l*_1_ optimization problem in Eq 4 can be generalized as follows:

$$\text{min}\left\{\frac{1}{2}\|y-Au(x,\alpha ){\|}_{2}^{2}+\lambda \sum_{i=1}^{n}\|\alpha_{i}{\|}_{1}\right\}$$ (19)

where *n* is the number of level-set functions.

The procedure for determining the coefficients and obtaining the optimal solution using the proposed method is summarized in the following algorithm:

**Algorithm 1** Reconstruction Algorithm

**Input:** $A\in {\mathbb{R}}^{M\times N}$, $y\in {\mathbb{R}}^{M}$, *u*_0_, *u*_1_, $\alpha_{0}$, *M*

**Output:** α, *u*

1. For *k* = 0 to itermax do
  (a) Compute the PLS $\varphi (x,\alpha_{k})$ from Eq 16
  (b) Calculate the Heaviside function $H(\varphi (x,\alpha_{k}))$ using Eq 13
  (c) Compute the model $u(x,\alpha_{k})$ from Eq 15
  (d) Compute $f(\alpha_{k})=\frac{1}{2}\|y-Au(x,\alpha_{k}){\|}_{2}^{2}$
  (e) Compute the gradient from Eq 25
  (f) Update $\alpha_{k+1}=\text{OWL-QN}(f(\alpha_{k}),\nabla f(\alpha_{k}),\alpha_{k})$
2. Set $\alpha =\alpha_{k+1}$
3. Compute *u* from Eq 15.

In the context of multiphase level-set functions, our approach differs fundamentally from the Sparse Shape Composition (SSC) framework proposed in [30]. SSC enables shape formation through set operations—such as unions and intersections—of predefined shape primitives, and promotes sparsity via an 𝚤_1_-norm constraint on dictionary coefficients. While this allows for flexible geometric shape modeling, SSC suffers from a significant limitation: the 𝚤_1_-norm constraint in high-dimensional spaces leads to a large feasible solution space, increasing the risk of inaccurate or unstable reconstructions.

Our method targets discrete gray-level recovery in tomographic reconstruction and employs a unified dictionary composed of single- and multi-scale Gaussian basis functions shared across all level-set functions. In contrast to SSC, which constructs and combines shapes using separate dictionaries for each level-set function, our method utilizes a common, shared basis across all phases. This unified representation reduces model complexity, avoids over-parameterization, and ensures consistent feature representation across levels. By enforcing sparsity through 𝚤_1_-regularization over this shared dictionary, the proposed framework achieves enhanced numerical stability, improved boundary preservation, and robust performance in both sparse-view and limited-angle CT scenarios. Consequently, Eq 16 can be expressed in a more general form:

$$\varphi (x,\alpha )=\sum_{j=1}^{n}\sum_{i=1}^{N}(\alpha_{j}{)}_{i}m_{i}(x).$$ (20)

To solve Eq 19, an iterative process is required, including the computation of the gradient of the cost function at each iteration. Assuming:

$$f(\alpha )=\frac{1}{2}\|y-Au(x,\alpha ){\|}_{2}^{2}$$ (21)

For the case of a single level set, differentiating Eq 15 with respect to α yields:

$$\frac{\partial u}{\partial \alpha }=M\left((u_{1}-u_{0})\delta (\varphi -c)\right)$$ (22)

where δ(·) denotes the Dirac delta function [27].

For multiphase level sets, differentiating Eq 18 with respect to $\alpha_{1}$ and $\alpha_{2}$ yields:

$$\frac{\partial u}{\partial \alpha_{1}}=M\left((u_{1}-u_{0})\delta (\varphi_{1}-c)+(u_{2}-u_{1})\delta (\varphi_{1}-c)H(\varphi_{2}-c)\right)$$ (23)

$$\frac{\partial u}{\partial \alpha_{2}}=M\left((u_{2}-u_{1})H(\varphi_{1}-c)\delta (\varphi_{2}-c)\right)$$ (24)

From Eq 21, the derivatives of the function f(α) can be expressed as:

$$\frac{\partial f(\alpha )}{\partial \alpha }=\sum_{j=1}^{n}\frac{\partial f(\alpha )}{\partial \alpha_{j}}=\frac{\partial f}{\partial u}\sum_{j=1}^{n}\frac{\partial u}{\partial \alpha_{j}}$$ (25)

## Basis function

Several options exist for selecting basis functions, including Gaussian functions, multi-quadric functions, polyharmonic splines, and thin plate splines. In this paper, we focus on the Gaussian function (GF) due to its flexibility and straightforward representation. The Gaussian function is given by:

$$m_{i}(x)=\exp\left(-\frac{\|x-x_{i}{\|}^{2}}{2\gamma^{2}}\right),$$ (26)

where ‖ · ‖ denotes the Euclidean norm, *x*_*i*_ represents the center of the GF, and γ is the width of the GF. This can be rewritten as:

$$m_{i}(x)=\exp(-\beta \|x-x_{i}{\|}^{2}),$$ (27)

where

$$\beta =\frac{1}{2\gamma^{2}}.$$ (28)

Two crucial factors influence the representation power and recovery accuracy of the proposed method:

1. The number of GFs in the dictionary.
2. The value of the shape parameter γ.

The selection of Gaussian centers and the shape parameter for GF in parametric level set (PLS) reconstruction will be discussed in the following sections.

### Distribution of the GF centers

The placement of GF centers significantly impacts the accuracy of recovering fine details in the reconstructed image. Previous studies [35, 36] indicated that placing GFs on a lattice grid in the image domain can affect recovery accuracy.

Historically, radial basis function (RBF) centers were placed arbitrarily, either on regular or irregular grids, depending on the specific application [26, 28], and [37]. For instance, [37] improved center placement by using image features or prior shape information. They compared two scenarios: using a regular grid when no prior knowledge was available and distributing centers near the boundary when prior shape information was available, concluding that center placement directly impacts accuracy.

Building on these insights, our approach adopts a distinct strategy. We use a large number of GFs, equal to the number of pixels in the domain, creating a dictionary where each pixel has its own GF. To manage the large dictionary size, we utilize the 𝚤_1_-norm and impose constraints on the image’s gray levels within our cost function. This approach efficiently removes unused centers and minimizes the cost function, and it performs well across various images with different topologies without requiring different center distributions for different applications.

### Shape parameter

The shape parameter γ (or equivalently $\beta =\frac{1}{2\gamma^{2}}$) controls the spatial extent of each Gaussian basis function and plays a crucial role in balancing reconstruction quality, sparsity, and numerical stability. A smaller γ leads to narrower functions that can capture fine details, but may cause overfitting, slower convergence, and ill-conditioned optimization. In contrast, a larger γ results in wider functions that improve smoothness and convergence but may reduce spatial accuracy. Based on prior studies [35, 36] and our empirical tests, we select γ∈[0.1,0.5] as a practical trade-off between sparsity, numerical stability, and reconstruction quality. Although we use a fixed γ to simplify implementation and reduce computation, future work could explore adaptive or learned shape parameters to enhance flexibility across varying image features or modalities.

To optimize the basis function width, we consider two approaches: a constant width for all Gaussian functions, and an estimated width for each one. Given our distribution strategy, we adopt the constant width approach to reduce computational cost while maintaining stable performance.

**Fig 3:**
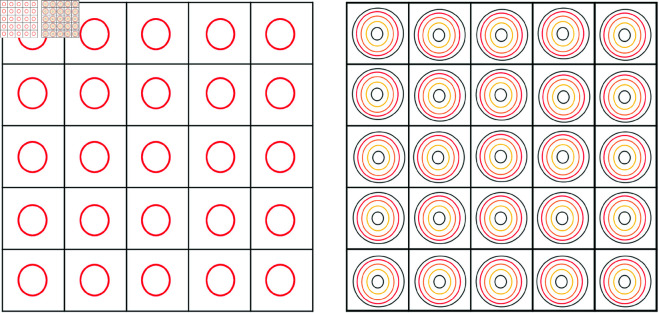
This figure Illustrates the placement of single-scale and multiscale Gaussian Functions at each pixel, represented as circles.

Our research utilizes both single-scale and multiscale Gaussian functions for image representation. The spatial distribution of these functions is illustrated in Fig 3.

- **Single-Scale GF:**

In this approach, we empirically determine an optimal value of β (corresponding to a fixed γ) and construct a dictionary consisting of one Gaussian function centered at each image pixel.

- **Multiscale GF:**

To improve sparsity and capture multi-resolution features, we employ a multiscale Gaussian function strategy. Specifically, we use ten different values of γ within the range [0.1,0.5], resulting in a richer dictionary composed of ten Gaussian functions per pixel.

## Experimental studies

In this section, we present numerical results that showcase the performance, robustness, and accuracy of our proposed method for limited data X-ray tomography. We conducted experiments using both numerical phantoms and an experimental X-ray dataset, evaluating both single-level-set and multiphase-level-set functions. Additionally, we compared our method against several state-of-the-art iterative reconstruction techniques, including the Total Variation (TV) method, which involves solving an optimization problem using the Chambolle-Pock algorithm with non-negativity constraints [38]; the DART [19]; and the Dual Problem (DP) approach [39].

For fair comparison, we implemented TV, DART, and DP using consistent parameter choices and stopping criteria across all experiments. For the TV method, we used a regularization parameter λ=8 × 10^−3^, tuned empirically, with convergence defined by a tolerance of 10^−6^ on both optimality and progress. DART was configured to run for 50 iterations, with grayscale levels ρ={0,100} and a segmentation threshold τ=30. The DP method followed its reference implementation, running until the optimality gap fell below 10^−6^ or the maximum number of iterations was reached. All methods were executed either until convergence or until a fixed iteration limit.

To comprehensively assess the proposed method, we evaluated its performance in sparse-view and limited-angle scenarios, along with its robustness to noise. Gaussian noise was incorporated into the projection data at varying levels (e.g., n=0,n=0.01,n=0.05, and *n* = 0.1), simulating real-world conditions. Reconstructions were performed for both sparse-view and limited-angle configurations, with noise robustness assessed quantitatively using PSNR and SSIM metrics.

**Fig 4:**
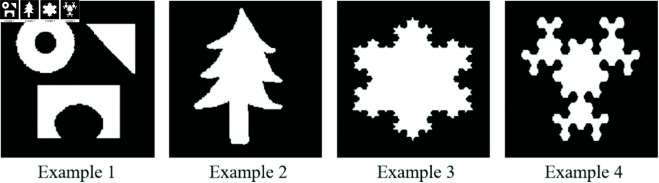
Synthetic images used in simulation experiments to assess the performance of the proposed method.

To assess the effectiveness of our method, we performed synthetic tests on four different examples, each featuring distinct shapes as illustrated in Fig 4. The synthetic shapes shown in Fig 4 were deliberately designed to test specific capabilities of the reconstruction algorithms, including edge preservation, shape regularity, discrete segmentation, and reconstruction of non-convex or disconnected features. This variety ensures a thorough evaluation of each method’s robustness in handling geometric complexity and data incompleteness.

**Fig 5:**
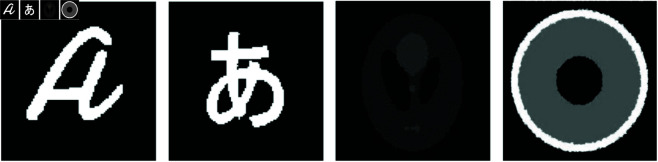
Two letters and two images with three different gray levels were used in the simulation experiments to evaluate the performance of the proposed method.

We also evaluated our approach with two different characters, depicted in Fig 5, using gray levels set to *u*_0_ = 0 and *u*_1_ = 1. For the multiphase-level-set scenario, we examined two images with three different gray levels, as shown in Fig 5.

### Evaluation metrics

To evaluate the accuracy of our simulation results and to compare the performance of the proposed method with existing techniques, we conducted assessments under sparse view and limited angle conditions. We utilized three widely accepted image quality metrics: the Peak Signal-to-Noise Ratio (PSNR), the Structural Similarity Index Measure (SSIM) [40, 41], and the Dice coefficient [42].

The PSNR is defined as:

$$\text{PSNR}=10\cdot \log_{10}\left(\frac{{\text{MAX}}^{2}}{\text{MSE}}\right),$$ (29)

where MAX represents the maximum possible pixel value in the image, and MSE denotes the Mean Squared Error between the original image (*x*) and the reconstructed image (${\text{x}}^{*}$):

$$\text{MSE}(x,x^{*})=\frac{1}{MN}\sum_{i=0}^{M-1}\sum_{j=0}^{N-1}(x(i,j)-x^{*}(i,j){)}^{2},$$ (30)

where *M* and *N* are the dimensions of the image, and *x*(*i*,*j*) and ${\text{x}}^{*}(\text{i},\text{j})$ are the pixel values of the original and reconstructed images, respectively.

The SSIM is defined as:

$$\text{SSIM}(x,x^{*})=\frac{\left(2\mu_{x}\mu_{x^{*}}+c_{1})(2\sigma_{x,x^{*}}+c_{2}\right)}{\left(\mu_{x}^{2}+\mu_{x^{*}}^{2}+c_{1})(\sigma_{x}^{2}+\sigma_{x^{*}}^{2}+c_{2}\right)},$$ (31)

where $\mu_{x}$ and $\mu_{x^{*}}$ represent the mean values of *x* and ${\text{x}}^{*}$, respectively. $\sigma_{x}^{2}$ and $\sigma_{x^{*}}^{2}$ denote the variances, and $\sigma_{x,x^{*}}$ is the covariance. The constants *c*_1_ and *c*_2_ are used to stabilize the division with weak denominators.

To complement these pixel-level and structural metrics, we also report the Dice coefficient, which is particularly relevant in segmentation-like reconstruction tasks where the accuracy of region boundaries is critical. The Dice coefficient is defined as:

$$\text{Dice}(A,B)=\frac{2|A\cap B|}{\left|A|+|B\right|}$$ (32)

where *A* and *B* represent the sets of foreground pixels in the ground truth and reconstructed images, respectively. Higher Dice values indicate better overlap and region reconstruction.

Table 1 summarizes Dice scores for various methods under sparse-view and limited-angle conditions. The proposed method consistently achieves the highest Dice values across all test configurations, confirming its strength in accurately recovering region boundaries and discrete intensity levels.

**Table 1:** Comparison of reconstruction accuracy under sparse-view and limited-angle scenarios using different methods. Values represent Dice coefficients (higher is better).

Method	Sparse view			Limited angle using 8 projections		
	4 proj	6 proj	8 proj	π/2	7π/12	5π/6
**TV**	0.5270	0.5610	0.5870	0.5660	0.5640	0.5630
**DART**	0.7167	0.7415	0.7469	0.7024	0.7268	0.7520
**PALS**	0.8980	0.8810	0.8880	0.8820	0.8500	0.9020
**Ours**	0.9550	0.9710	0.9670	0.9670	0.9580	0.9630

While PSNR and SSIM provide standard measures of global reconstruction quality, they do not fully capture boundary sharpness or region-specific accuracy, which are crucial in discrete tomography. Region-based metrics such as Dice or confusion matrices may favor smoother reconstructions like TV that suppress noise but blur edges. Our method prioritizes precise boundary delineation and discrete level recovery, which are better appreciated through qualitative visual inspection and edge preservation analysis.

### Tests

We conducted two distinct tests to evaluate the robustness of the proposed method in addressing limited data problems. Firstly, we investigated the impact of sparse projection data. Sparse sampling aims to decrease scan time by reducing the number of projection angles used. In our experiments with synthetic images, we started with 10 projections distributed across the range [0,π], then progressively reduced the number of projections to 6 and 4 for various examples to assess performance under increasingly sparse conditions.

Secondly, we explored the limited-angle problem, where projections were acquired within a restricted range $\left[0,\theta_{\text{max}}\right]$. The values of $\theta_{\text{max}}$ considered were {5π/6,2π/3,7π/12,π/2}. This scenario often results in streak artifacts in the reconstructed images. Our goal was to demonstrate how our proposed method effectively mitigates these artifacts, thereby improving image quality.

As the angular range narrows, reconstruction degradation becomes more pronounced, particularly in methods like TV and DP that rely on more complete angular coverage. Our method remains robust across angular reductions by leveraging the sparsity and localization of Gaussian basis functions, which help maintain edge contrast even under incomplete sampling. The performance trends in later sections show that our method degrades more gracefully compared to competing approaches.

### Real data

**Fig 6:**
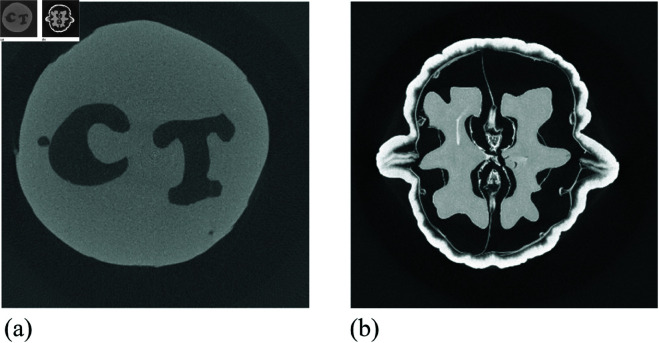
High-resolution reconstruction using FBP for (a) a carved cheese slice from 360 projections, and (b) a walnut slice from 1200 projections.

To evaluate the performance of our proposed method, we conducted experiments using real X-ray CT projection data for both single-level-set and multiphase-level-set functions. In the single-level-set scenario, we used X-ray projection data of a carved cheese slice [43]. Fig 6(a) presents a high-resolution filtered back-projection reconstruction from this data, obtained using a fan-beam sinogram with a resolution of 2000 × 2000 and 360 projections. The dataset includes two distinct gray levels corresponding to the cheese slice and the surrounding air. For sparse view experiments, we used DataFull_128×15.mat, containing 15 projections across a 360-degree rotation. For the limited-angle case, we used DataLimited_128×15.mat, which provides 15 projections over a 90-degree range.

In the multiphase-level-set scenario, we analyzed tomographic X-ray data of a walnut slice [44]. Fig 6(b) shows a high-resolution FBP reconstruction from 1200 projections (2296×2296 resolution). This dataset reveals three distinct gray levels representing the walnut’s shell, kernel, and air. We used Data164.mat, containing sinogram data from 164 fan beams at 120 angles, with reconstruction resolution of 164×164.

### Sparse view CT

**Fig 7:**
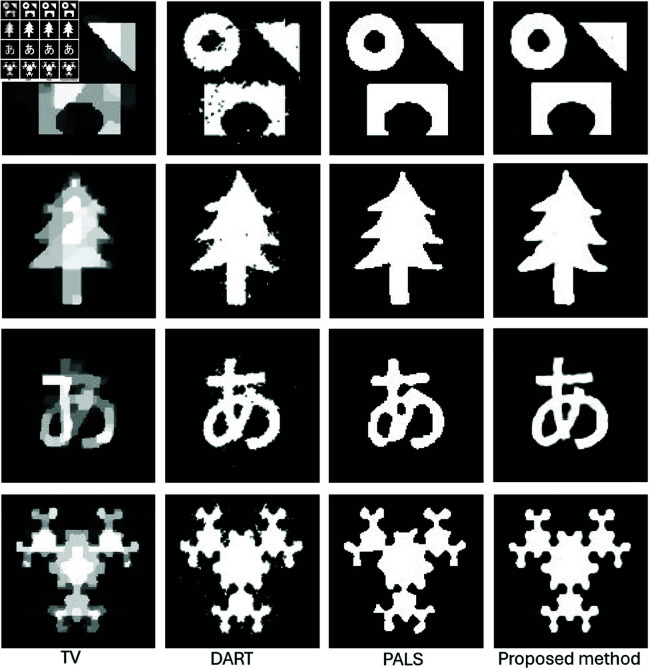
Performance comparison of the proposed method against various reconstruction techniques using data from only four projections for the first two tests and six projections for the last two tests.

We evaluated the performance of our proposed method by reconstructing images from a limited number of projections within the angular range [0,π]. Fig 7 illustrates the results for four different examples. For the first and second examples, we used only four projections, while the third and fourth examples with fine details and complex topologies were reconstructed using six projections.

**Fig 8:**
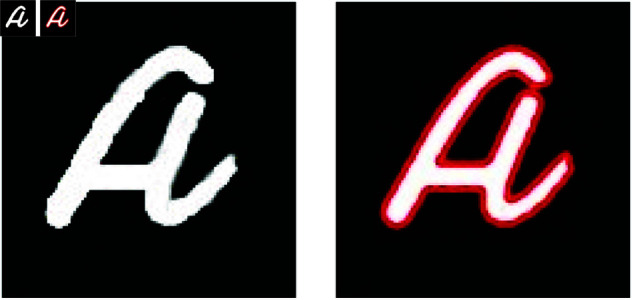
Testing the proposed method for character reconstruction using 6 projections within the angular range [0,π].

The results demonstrate that our method significantly outperforms traditional techniques by delivering high-quality images with well-defined boundaries, even with few projections. In contrast, methods such as TV and DART exhibit noticeable artifacts due to data insufficiency. The parametric level set method (PALS) from [26], using Wendland-4 RBFs with wider spacing, shows satisfactory results in simpler cases but struggles with complex shapes (Fig 7). As projection count decreases, competing methods show degraded quality, while our method maintains accurate reconstructions using strategically placed Gaussian functions and optimized shape parameters (Figs 7 and 8).

**Fig 9:**
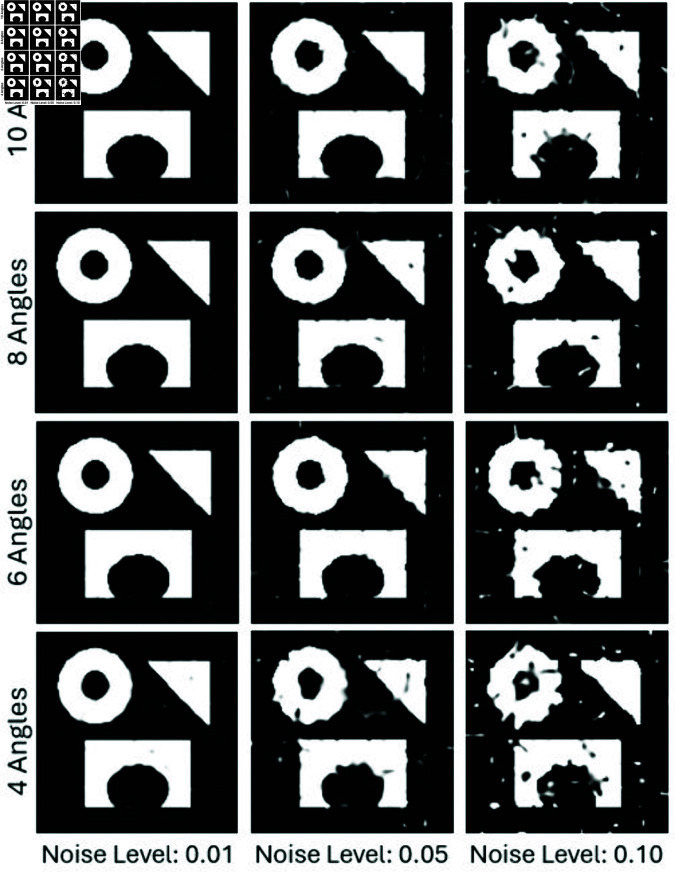
Reconstruction results using projection data from 4, 6, 8, and 10 angles under noise levels of n = 0.01, 0.05, and 0.10.

**Fig 10:**
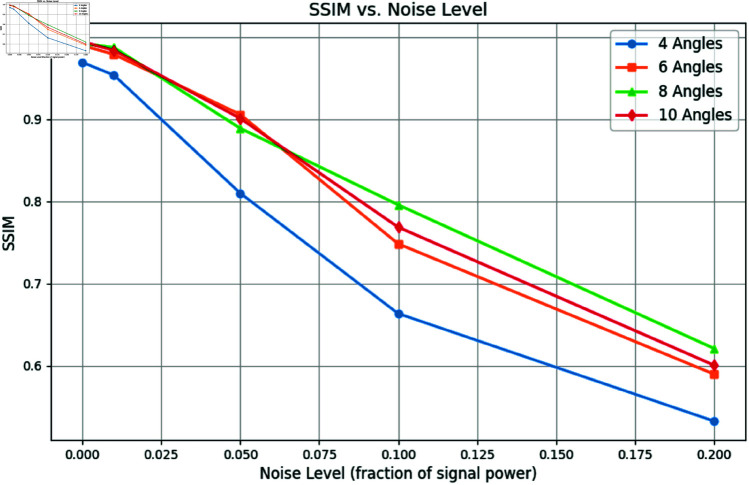
SSIM vs. Noise Level: Reconstruction quality comparison using 4, 6, 8, and 10 projection angles.

**Fig 11:**
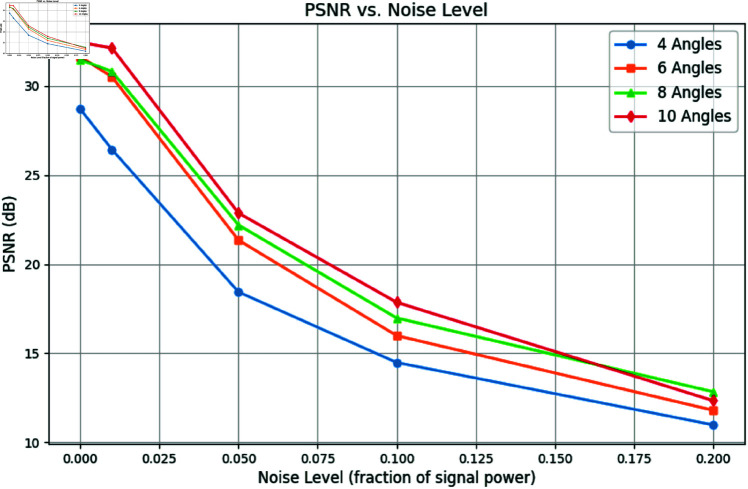
PSNR vs. Noise Level: Reconstruction quality comparison using 4, 6, 8, and 10 projection angles.

To further evaluate the robustness of the proposed method under real-world conditions, we incorporated varying levels of Gaussian noise into the projection data. For this test, we focused on one example and reconstructed images using 4, 6, 8, and 10 projections under noise levels *n* = 0.01, *n* = 0.05, and *n* = 0.1 as shown in Fig 9. Figs 10 and 11 show the SSIM and PSNR trends for these tests, highlighting the method’s ability to maintain reconstruction quality across varying noise levels. The quantitative results demonstrate that as the number of projections increases, the method becomes more robust to noise, achieving a PSNR of 22.87dB and an SSIM of 0.901 for 10 projections at *n* = 0.05.

**Fig 12:**
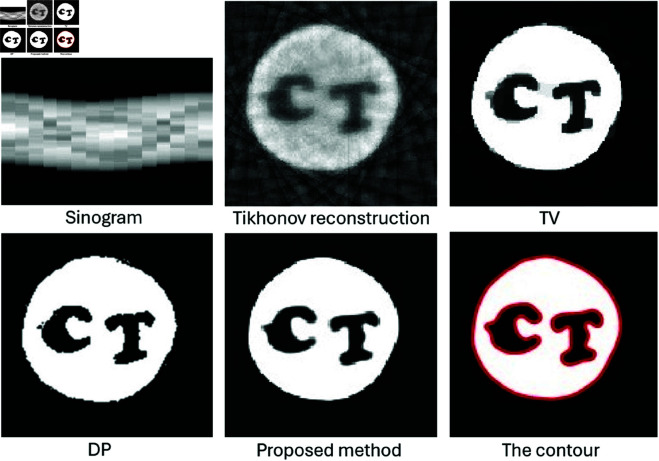
Performance comparison of the proposed method with Tikhonov, Total Variation (TV), and Dual Problem (DP) reconstructions on a real CT dataset of a carved cheese slice with sparse projections. The sinogram was acquired from 15 projections over a 360^∘^ range.

In addition to synthetic data evaluation, we tested our method on real computed tomography (CT) data. Fig 12 showcases the results from a carved cheese slice dataset with two materials. The sinogram, acquired from 15 projections over a 360-degree range, along with the reconstructions from our method and comparison techniques, highlights that while TV and DP methods approximate the ground truth, our method excels in delineating the carved cheese slice with smooth, precise boundaries and successfully identifying small features such as letters and holes.

**Fig 13:**
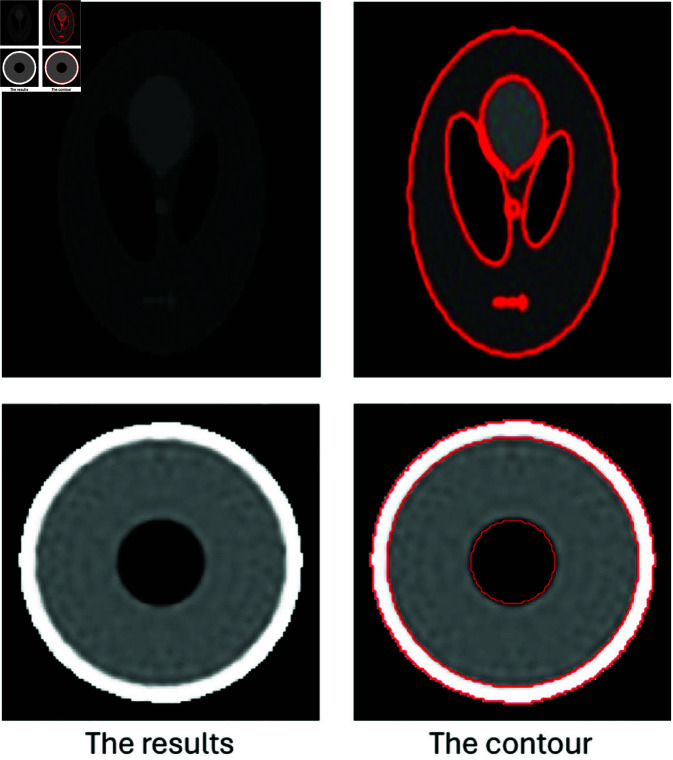
Performance of the proposed method for multiphase level-set functions.

**Fig 14:**
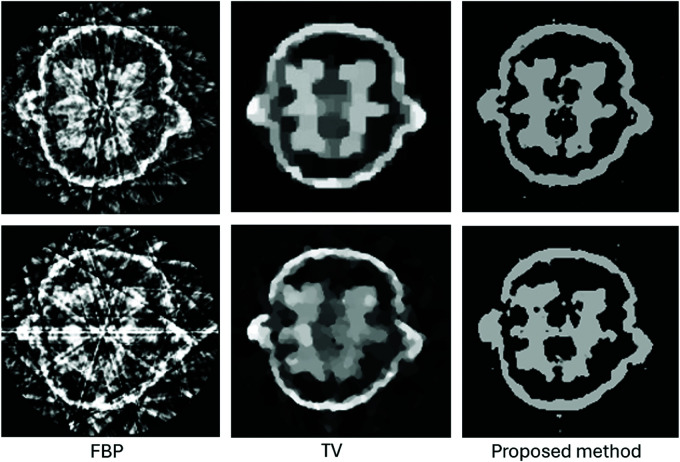
Comparison of reconstruction methods on real CT data of a walnut slice using 20 projections (top row) and 10 projections (bottom row). From left to right: Filtered Back Projection (FBP), Total Variation (TV) regularization, and the proposed method.

For evaluating multiphase level set functions, we tested the proposed method using two synthetic images, as shown in Fig 5. Each image consists of three distinct regions, and we employed two level set functions to represent these regions effectively. Fig 13 demonstrates our method’s performance in reconstructing these images, showcasing its ability to accurately delineate the boundaries of each region and handle complex, multiphase structures. We further applied our method to a real CT scan of a 2D walnut slice with three distinct materials. Fig 14 compare our reconstruction results with fbp and TV reconstruction method described in [38], using sinogram obtained from fan beams at 20 and 10 angles. As observed in the walnut dataset, the TV scheme achieves better homogeneity in some regions. However, the proposed method excels in accurately delineating fine boundaries, which is critical for discrete tomography. Fig 14 highlights the trade-offs between regional smoothness and boundary accuracy. These results suggest that the proposed method is particularly well-suited for applications requiring precise boundary reconstruction under sparse and limited-angle conditions.

To quantitatively assess performance, we used PSNR and SSIM metrics, as detailed in Table 2. The proposed method consistently achieves the highest PSNR and SSIM values across various projection scenarios, indicating superior reconstruction quality. Furthermore, the results demonstrate that increasing the number of projections improves both PSNR and SSIM scores, reflecting enhanced reconstruction fidelity and structural accuracy.

**Table 2:** Performance metrics (PSNR and SSIM) for different reconstruction methods across different sparse views.

Angles	Example	TV		DART		PALS		Proposed Method	
		PSNR	SSIM	PSNR	SSIM	PSNR	SSIM	PSNR	SSIM
10	E1	35.9510	0.9912	34.9485	0.9940	31.0947	0.9924	38.4585	0.9963
	E2	33.0711	0.9901	21.9397	0.8935	30.4841	0.9916	40.7327	0.9982
	E3	27.3113	0.9777	24.4563	0.9619	18.5256	0.8877	42.2357	0.9970
	E4	18.1044	0.6057	30.1773	0.9902	21.5290	0.9465	37.8495	0.9953
8	E1	35.9533	0.9890	34.1567	0.9931	30.6365	0.9919	38.2201	0.9963
	E2	30.4514	0.9719	21.5027	0.8861	30.8492	0.9924	39.2750	0.9965
	E3	26.5096	0.9674	22.6954	0.9403	17.6006	0.8715	38.8222	0.9930
	E4	20.7514	0.7776	25.7057	0.9774	21.3745	0.9452	34.7040	0.9953
6	E1	27.3113	0.9777	32.3958	0.9903	31.2169	0.9916	34.6569	0.9891
	E2	21.1827	0.7587	21.4700	0.8811	29.3117	0.9891	39.5503	0.9957
	E3	24.9852	0.9511	20.5081	0.8874	18.0319	0.8771	35.4511	0.9820
	E4	16.0089	0.5512	16.7399	0.8045	21.0082	0.9371	27.9005	0.9655
4	E1	18.1044	0.6057	16.5853	0.7397	28.4872	0.9840	28.9696	0.9650
	E2	16.5155	0.6276	18.9365	0.7940	26.6429	0.9796	30.1657	0.9727
	E3	15.4103	0.3737	18.8847	0.8532	17.5131	0.8628	22.9858	0.8504
	E4	11.4655	0.1432	10.7821	0.5466	11.9385	0.7164	15.0503	0.7044

In the sparse projection case with 4 angles, although PALS achieves a slightly higher SSIM compared to the proposed method, this comes at the cost of significantly reduced PSNR. This difference arises from PALS’s tendency to apply aggressive smoothing during reconstruction, which inflates SSIM values by suppressing noise at the expense of detail preservation. In contrast, the proposed method prioritizes boundary sharpness and accurate modeling of discrete intensity levels. These strengths make it particularly well-suited for discrete tomography, where reconstruction fidelity and boundary accuracy are prioritized over marginal SSIM improvements.

### Limited-angle CT

**Fig 15:**
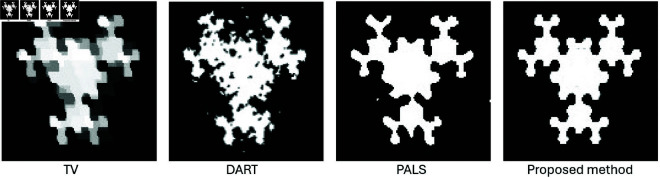
Performance of various reconstruction methods in a limited-angle test using 8 projections spanning from 0 to π/2.

We also assessed the performance of the proposed method under limited-angle conditions, we conducted experiments using various angular ranges. Fig 15 shows the results for Example 4, where different reconstruction methods were compared using only 8 projections within the range of 0 to π/2. The proposed method yields reconstructions that closely match the true images and surpass other methods in quality.

**Fig 16:**
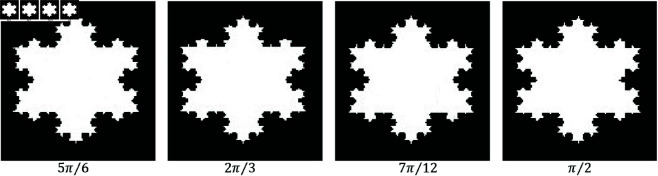
Performance of the proposed method across different angular ranges using 8 projections.

Fig 16 illustrates reconstructions of Example 3 under various angular ranges. Despite narrowing angles, the proposed method preserves structure and edge detail better than alternatives.

**Fig 17:**
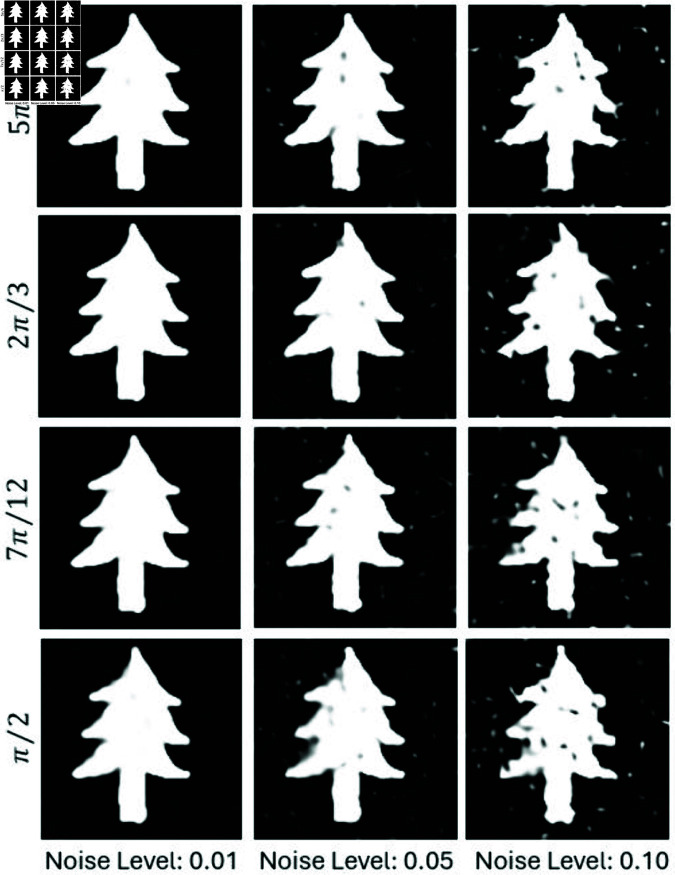
Reconstruction results vs. noise levels using limited projection data from 0 to π/2, 7π/12, 2π/3, and 5π/6.

**Fig 18:**
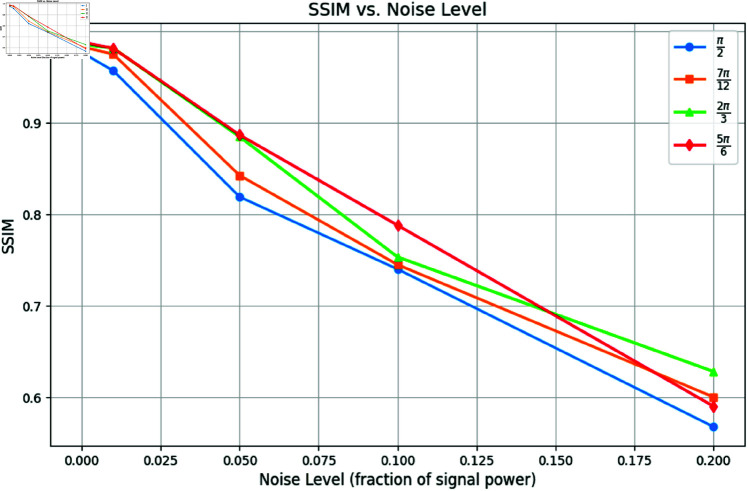
SSIM vs. Noise level: Reconstruction quality comparison using limited projection data from 0 to π/2, 7π/12, 2π/3, and 5π/6.

**Fig 19:**
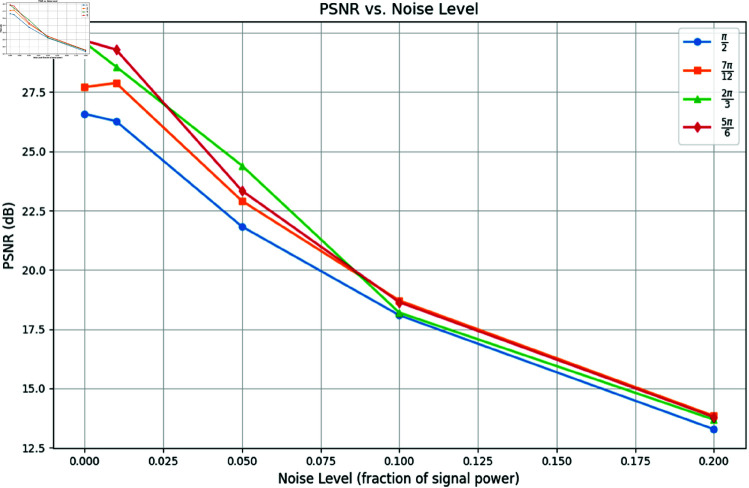
PSNR vs. Noise level: Reconstruction quality comparison using limited projection data from 0 to π/2, 7π/12, 2π/3, and 5π/6.

We further tested robustness by adding Gaussian noise at three levels and using 10 projections within angles {π/2,7π/12,2π/3,5π/6} (Fig 17). Figs 18 and 19 show PSNR and SSIM trends. Our method maintains high performance even under severe limitations.

**Fig 20:**
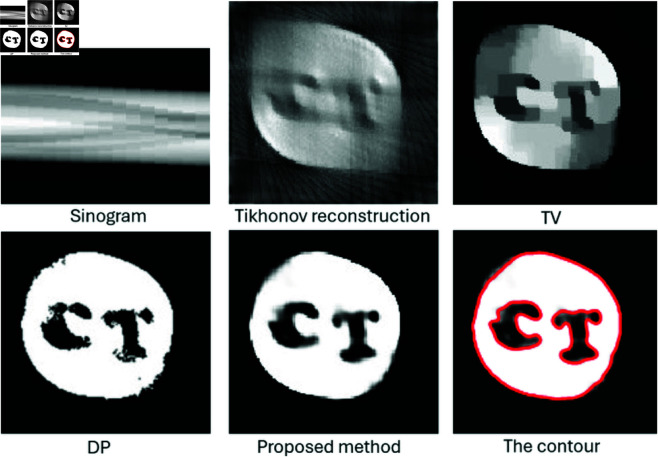
Performance comparison of the proposed method with Tikhonov, Total Variation (TV), and Dual Problem (DP) reconstructions on a real CT dataset of a carved cheese slice under limited-angle tomography. The sinogram was obtained from 15 projections spanning an angular range from 1 to π/2.

For real data testing, we used the same carved cheese slice dataset with limited projections from 0 to π/2. Fig 20 displays the sinogram obtained from 15 projections spanning 1 to π/2, alongside the reconstruction results from three different methods compared to our proposed method. The Tikhonov and TV reconstructions show streak artifacts and blurring in the carved regions due to the limited data. Although the DP method avoids these artifacts, it still fails to accurately delineate the region’s boundary. In contrast, our method accurately captures the shape of the cheese and correctly identifies the regions labeled C and T.

Table 3 provides quantitative metrics for the TV, DART, PALS, and proposed methods under limited-angle conditions, using 8 projections across different angular ranges. The proposed method demonstrates the best performance in these evaluations, indicating that its reconstruction results are closest to the ground truth. This performance highlights the method’s superior artifact reduction, edge preservation, and noise suppression capabilities.

**Table 3:** Performance metrics (PSNR and SSIM) for different reconstruction methods under limited-angle views using only 8 projections across various angular ranges.

Angles	Example	TV		DART		PALS		Proposed Method	
		PSNR	SSIM	PSNR	SSIM	PSNR	SSIM	PSNR	SSIM
5π/6	E1	29.7928	0.9187	29.3855	0.9745	29.6063	0.9897	37.2891	0.9925
	E2	25.8905	0.9389	23.2035	0.9122	31.0380	0.9923	39.9461	0.9958
	E3	23.4173	0.9373	25.4060	0.9763	19.0279	0.8883	37.1205	0.9838
	E4	15.7655	0.5442	19.9516	0.8894	19.6819	0.9122	27.3602	0.9582
2π/3	E1	27.3404	0.9071	31.5243	0.9883	32.2491	0.9948	37.7234	0.9936
	E2	25.0717	0.9083	23.5904	0.9182	28.7420	0.9870	40.9517	0.9975
	E3	24.6266	0.9578	30.1773	0.9903	19.1217	0.8908	39.5937	0.9922
	E4	13.8764	0.4130	20.3848	0.8819	18.3058	0.8670	21.6468	0.8561
7π/12	E1	23.5859	0.8631	30.4769	0.9826	31.6612	0.9930	38.1380	0.9963
	E2	22.9148	0.9004	23.2892	0.9140	28.1836	0.9845	38.5825	0.9968
	E3	25.7561	0.9578	26.1404	0.9745	18.3412	0.8812	36.4628	0.9810
	E4	15.7141	0.5306	18.6138	0.8677	19.9067	0.9181	30.2365	0.9723
π/2	E1	20.3674	0.8319	23.9448	0.9384	31.5839	0.9933	35.2760	0.9911
	E2	19.4891	0.7585	22.4864	0.9069	28.9203	0.9880	37.2760	0.9936
	E3	25.9704	0.9709	24.5346	0.9623	18.4907	0.8867	35.2927	0.9725
	E4	17.6181	0.6577	15.2637	0.7679	20.4325	0.9262	33.9712	0.9849

## Conclusion

In this study, we introduced a novel reconstruction framework for discrete tomography that integrates compressed sensing (CS) with the parametric level set (PLS) method. The proposed approach is designed to address the challenges posed by sparse-view and limited-angle geometries by leveraging prior knowledge of discrete gray-level values in the target image.

Our method enforces sparsity through *l*_1_-norm regularization on a dictionary of Gaussian basis functions, which are assigned at each pixel and constructed using either single-scale or multiscale strategies. This formulation allows for a compact, compressible representation of image boundaries and internal structures. To efficiently solve the resulting optimization problem, we adopted the Orthant-Wise Limited-memory Quasi-Newton (OWL-QN) algorithm, which demonstrated reliable convergence and reconstruction accuracy.

Comprehensive numerical simulations and experiments using real CT data confirmed the effectiveness of the framework. The method consistently produced high-quality reconstructions in both sparse-view and limited-angle scenarios, with strong resilience to Gaussian noise. Quantitative metrics, including PSNR, SSIM, and Dice coefficients, highlighted the advantages of the method in preserving fine boundary structures and accurately recovering discrete intensity levels.

When compared with state-of-the-art techniques such as Tikhonov regularization, Total Variation (TV), DART, PALS, and Dual Problem (DP) methods, our framework achieved superior reconstruction performance, particularly in artifact reduction and boundary sharpness. While current results are promising, future work will investigate adaptive dictionary selection, 3D extensions, and real-time implementations to further enhance applicability in clinical and industrial imaging settings.
